# Characteristics as Taught by Hahnemann

**Published:** 1888-09

**Authors:** Edmund J. Lee


### HOMŒOPATHIC PHYSICIAN,

A MONTHLY JOURNAL OF

HOMŒOPATHIC MATERIA MEDICA AND CLINICAL MEDICINE.

"If our school ever give up the strict inductive method of Hahnemann, we are lost, and deserve only to be mentioned as a caricature in the history of medicine."—CONSTANTINE HERING.

Vol. VIII. SEPTEMBER, 1888. No. 9.

### CHARACTERISTICS AS TAUGHT BY HAHNEMANN.

In considering "Characteristics as taught by Hahnemann," we have for our guide such parts of the *Organon* as treat of this subject, also a few notes and cases left by Hahnemann. Though Hahnemann's teaching upon this part of homœopathic practice is clear and unmistakable as far it goes, yet we would be the wiser had we more of his sound and practical advice. The whole purpose of our present consideration of these "characteristics" is to ascertain what part they are to have in aiding us to select the simillimum.

The work of selecting the homœopathic curative is a two-fold one, for we have to study the symptoms of the patient on the one hand, and on the other the drug provings from whic we are to choose our remedy. As for the patient, we are t select from the results of a careful examination those symptom which are peculiar to the *individual* under treatment; this will necessarily exclude those symptoms which are diagnostic of th disease. These diagnostic symptoms are almost always useles as an aid to prescribing, for they are to be found in every similar case of disease. As to the drug, Hahnemann tells us, in par. 118: "Each medicine produces particular effects in the body of man, and no other medicinal substance can create any that are precisely similar." In order to prescribe these drugs accurately, then, it is our duty to learn just what are the

"particular effects" of each remedy, that we may readily distinguish each drug from all the others.

This paragraph, on a casual glance, seems to give a false impression, for one can hardly recall a single symptom of any drug which is not to be found in the record of another drug! But on a closer examination of Hahnemann's words we find they *are correct;* he states that no two drugs produce "precisely similar" effects in the human body. A marked emphasis must be laid upon the words "*precisely similar*," for when Hahnemann's idea, as conveyed in these words, is understood, then his meaning becomes clear and his statement is found to be true. As an illustration, let us take the great polychrest, Sulphur; in the record of it as given by Dr. Lippe in his *Key to the Materia Medica*, we find for each and every symptom, a list of other remedies having similar symptoms. But none of these drugs have "precisely similar" symptoms; or to take single symptoms, as examples, we find the empty sensation in the abdomen so marked with Phosphorus under other drugs; so, too, the bearing down of Sepia is to be found with many other remedies, but these never occur as "precisely similar" effects.

As a further aid in learning just what these "particular effects" are, Hahnemann tells us, in par. 153: "* * * * * In this search for the homœopathic specific remedy (that is, in this comparison of the total signs of the natural sickness with the list of symptoms of available drugs, in order to find among these *one* bearing a pathogenetic power corresponding to and resembling the disease to be cured), the *striking*, *remarkable*, *uncommon*, and *peculiar* (characteristic) signs and symptoms of the case of sickness are to be especially and almost exclusively brought before the eye, for *these especially must be very like the drug that is being searched for in the symptom-lists* if this is to be the most suitable for the cure. The general and indefinite, such as loss of appetite, headache, weakness, restless sleep, discomforts, etc., if they are not more closely defined, *deserve little attention*, for one finds something about as indefinite in almost every sickness, and caused by almost every drug."

We are told the general and indefinite symptoms are useless because they are caused by almost every drug; these, then, are not among those symptoms which no two drugs produce in a "precisely similar" manner. The characteristic, that is, the "striking, remarkable, uncommon, and peculiar" symptoms are then the ones which no two drugs produce in a "*precisely similar*" manner, and these are the symptoms which are to be used in deciding on the simillimum. *The characteristic symp-*

*toms of any drug may be termed the particular effects of that drug which no other drug produces in a precisely similar manner.*

Hahnemann gives us yet another guide in the study of these characteristics, for he tells us that the mental symptoms are a surer guide to the proper selection than the pathological; he illustrates this by saying: "Aconite seldom or never effects a rapid and permanent cure when the temper of the patient is quiet and even; nor Nux vomica, when the disposition is mild and phlegmatic; nor Pulsatilla, when it is lively, serene, or obstinate; nor Ignatia, when the mind is unchangeable and little susceptible of either grief or fear." (Foot-note to page 187.) Why are the mental symptoms the most important ones? Because they are the most peculiar, the most striking and uncommon; because they have no pathological or diagnostic value. Mental symptoms seldom are of service in diagnosis; they are more indicative of the *individuality* of the patient than the physical symptoms.

We are also told in the *Organon* that the totality of the symptoms is to be our sole guide in the choice of the homœopathic simillimum; this, it would appear, is to be understood as meaning the *totality of the peculiar and uncommon symptoms*, for we have just been told that the others are useless, being found in almost every patient. Thus, in every prescription, the *totality of the peculiar symptoms* of the patient must be covered by the *characteristics of a drug;* these symptoms should be of equal importance in both cases. A characteristic symptom of a drug may be found in the history of a patient, but it may not be at all peculiar or characteristic of his sickness; some seemingly unimportant symptom may be much more so. Thus we should not prescribe Chamomilla for every baby that is quieted by being carried about in the arms; another symptom which Chamomilla has not may be much more peculiar and uncommon. A symptom may be very peculiar, as a concomitant to one disease and with another not at all so. Thus, for instance, a watery diarrhœa, with colic, would not be peculiar or uncommon, but as a concomitant of a bronchial catarrh it becomes a useful symptom, indicating, probably, Antimonium-tart. So, too, with such a symptom as "profuse micturition;" very many drugs have caused or have cured profuse micturition, hence this symptom alone, unqualified, is of little or no value in deciding one in the choice of his remedy. But if more closely qualified or defined this symptom may become very peculiar or uncommon; thus profuse micturition relieving a headache might indicate Gelsemium or Fluoric-acid; if the profuse micturition were accompanied by swelling of the feet and ankles, it would probably call for Eupatorium-per. The same symptom, occurring with diarrhœa, is found under Fluoric-acid and Agaricus; during profuse sweat, under Aconite, Antimonium-tart., Dulcamara, Phosphorus, and Thuja. So any common symptom may be qualified or defined so as make it a peculiar or characteristic one.

As one is able to discriminate between symptoms, judging when they are peculiar and uncommon and when they are common and useless—just so successful will he be in his prescribing. Returning to Chamomilla, for an example, we know that it is very common and general for babies to be quieted by being carried or rocked, therefore, a fretful baby so quieted presents nothing peculiar or remarkable. But a case of infantile convulsions so relieved is peculiar, and the condition becomes a valuable indication. Take other examples showing the necessity for this discrimination; Phosphorus has this well-known symptom: "As soon as water becomes warm in the stomach, it is thrown up." This is a characteristic symptom under Phosphorus, but as vomiting after drinking is found under some two dozen remedies, how are we to know when the vomiting is due to the "water becoming warm in the stomach," or when it occurs simply as a consequence of drinking? So again, nausea on smelling food, which is so characteristic under Colchicum, is to be found under at least two other drugs. Of course in practice we have no difficulty in deciding these questions after a reference to the accompanying symptoms, they are mentioned here simply to show the necessity for careful study of each case lest we mistake a common and general symptom for a characteristic one.

Characteristics, or key-notes, as they have been termed by the late Dr. Guernsey, have been misunderstood and misapplied; they should not be used as sole indicators for a remedy, but as guide posts, showing one the remedy to be studied.* Hahnemann calls the "striking, remarkable, uncommon, and peculiar" symptoms the characteristic ones. And in the few samples we have left of his prescribing, we find he makes a very careful analysis of all these symptoms; no one or two are picked out and a drug given for these only. We find no record of his having given a remedy because a symptom goes from right to left; or because a patient is restless, and goes from bed to chair, etc.;

---

*See, also, Dr. Berridge's article on "Key-notes and the Totality of the Symptoms," HOMŒOPATHIC PHYSICIAN, vol. VII, page 44.

or because red sand is found in the urine! Some very fine cures may have been made by such methods of prescribing, but they can be considered little less than chance hits. Secure a few symptoms from a patient which are really peculiar and uncommon with such disease, cover these by reliable symptoms which are characteristic of a drug, then one has a secure basis for his prescription.

We quote the notes of a case treated by Hahnemann, to show our readers the careful and painstaking manner in which he studied out his cases; it will be observed that each symptom is fully analyzed and related drugs well considered. CASE: "A washerwoman, over forty years old, had been sick more than three weeks, unable to pursue her avocation, when she consulted me.

"1. On any movement, especially at every step and worse on making a misstep, she has a shoot in the scrobiculis cordis, that comes, as she avers, every time from the left side.

"2. When she lies she feels quite well; then she has no pain anywhere, neither in side or scrobiculis.

"3. She cannot sleep after three o'clock in the morning.

"4. She relishes her food, but when she has eaten a little she feels sick.

"5. Then water collects in her mouth and runs out of it, like waterbrash.

"6. She has frequent empty eructations after every meal.

"7. Her temper is passionate, disposed to anger. Whenever the pain is severe she is covered with sweat. The catamenia were quite regular a fortnight since. In other respects, her health is good.

"Now as regards symptom 1, Belladona, China, and Rhus tox., cause shootings in the scrobiculis, but none of them only on motion, as is the case here. Pulsatilla certainly causes shootings in the scrobiculis on making a false step, but only as a rare alternating action, and has neither the same digestive derangements as occur here (symptom 4 compared with 5 and 6) nor the same state of disposition. Bryonia alone has among its chief alternating actions, as the whole list of its symptoms demonstrates, pains *from movement* and especially shooting pains, as also stitches, beneath the sternum (in the scrobiculis) on raising the arm, and on making a false step it occasions shooting in other parts.

"The negative symptom, 2, met with here answers especially to Bryonia. Few medicines (with the exception, perhaps, of Nux vomica and Rhus tox., in their alternating action, neither of which, however, are suitable for the other symptoms) show a complete relief to pains during rest and when lying; Bryonia does, however, in an especial manner. Symptom 3 is met with in several medicines, and also in Bryonia. Symptom 4 is certainly, as far as regards 'sickness after eating,' met with in several medicines (as Ignatia, Nux vomica, Mercurius, Ferrum, Belladonna, Pulsatilla, Cantharis, etc.), but in none so constantly and usually, nor with the relish for food, as in Bryonia.

"As regards symptom 5, several medicines certainly cause a flow of saliva, like waterbrash, just as well as Bryonia. The others, however, do not produce the remaining symptoms in a very similar manner; hence Bryonia is to be preferred to them in this point. Empty eructation (of wind only) after eating, symptom 6, is found in few medicines, and in none so constantly, so usually, and to such a degree, as in Bryonia.

"To number 7, one of the chief symptoms in diseases (see *Organon of Medicine*, par. 213), is the state of the disposition, and as Bryonia causes this (7) symptom also in an exactly similar manner, therefore Bryonia is for all these reasons to be preferred in this case to all other medicines."

This is a very mild and simple case, yet Hahnemann gave it his careful attention, thoroughly analyzing each important symptom, until finally all drugs, in any wise related to the case, are excluded and Bryonia left as the "most similar" remedy. A dose of this drug was given in a low potency, and the patient was able to work the next day.

Another instance of Hahnemann's analysis of drugs may be found in a letter he once wrote his friend, Stapf. The case was evidently one of those undeveloped cases, in which the symptoms do not clearly indicate any remedy, for it will be observed that Hahnemann suggests four remedies, one to follow the other as needed. Stapf had consulted Hahnemann about a patient, and mentioned Nux vom., Cham., China, and Puls., as best indicated. Hahnemann analyzed the case in this manner:

"Notwithstanding that Nux vom. produced perspiration standing on the forehead, perspiration when moving in general, perspiration during sleep; Chamomilla, perspiration especially about the head during sleep; Pulsatilla, perspiration during sleep, disappearing on awaking; China, perspiration when moving (crying), perspiration on the head especially (but also in the hair);—there is more indication for Pulsatilla by the itching of the eyes, which Puls. has, especially with redness in the *external* corner of the eye after rubbing, and with agglutination of eyelids in morning; if not, Ignatia would be preferable, which also cures itching and redness, but in the *internal* corners, with agglutination in the morning, in case the child's disposition is very changeable—now too lively, next peevishly crying, which Ignatia produces. If there should be, at the same time, a great sensitiveness to the daylight, when opening the eyes in the morning, which is also cured by Ignatia; or, in case of a mild disposition and a weeping mood in the evening, and a general aggravation of symptoms in the evening, Pulsatilla. The frequent awakening during the night indicates Ignatia more than Pulsatilla; the latter has more—a late falling asleep. The itching of the nose has been observed mostly from Nux vomica. Ignatia and Cham. have both, the latter more, pain *during* micturition; Pulsatilla the most pain before urinating. The loud breathing has been observed of China and Nux vom. —from the latter especially during sleep. As these remedies correspond much with each other (China excepted), and one corrects the faults and bad effects of the other (if only Ign. does not follow Nux v., or Nux v. is not given immediately after Ign., as they do not follow one another well on account of their great similarity), you can now judge as to the succession in which you may choose to employ Ign., Puls., Nux v., or Cham.—if the first, or one of the others, should not prove sufficient. To give Cham. there ought to be more thirst at night than at present and more irritability. China has little or nothing for itself, and is, therefore, not to be chosen."

Now, we have given three distinct statements, in which Hahnemann sums up his advice in regard to the art of homœopathic prescribing. These statements cover the whole ground, both as to the patient and the medicines. They are, briefly stated, thus:

1. Each medicine produces particular effects in the body of man, and no other medicinal substance can create any that are precisely similar.

2. In prescribing the striking, remarkable, uncommon, and peculiar symptoms of the patient are to be studied, for these must especially be covered by the characteristic symptoms of a remedy.

3. The totality of the symptoms is the only guide in the selection of the homœopathic remedy.

It will be observed that all the important words in these directions are in plural; thus we read of "particular effects" of drugs, of "peculiar symptoms," of the "totality of the symptoms." All indicating that in every case many factors are to be considered; in none do we read of single symptoms being used. Each drug has its particular symptoms, which, taken collectively, surely indicate that drug, but few, if any, drugs have one symptom which invariably calls for that remedy. Take Lycopodium, for instance, it has a group of symptoms, which, taken together, can always be relied upon to indicate that remedy, but each individual symptom of this group is to be found under other drugs. One could scarcely fail to know what remedy even these few symptoms called for. Aggravation from four to eight P. M.; symptoms going from right to left, especially of the throat; fan-like motion of alæ nasi; clear urine depositing a red sandy sediment; backache before urinating; a full, bloated feeling after eating a little; one foot hot, the other cold.

The whole art of prescribing, then, consists in finding, for each patient, that drug whose "particular effects" are most similar to the totality of the peculiar and uncommon symptoms exhibited by the patient. This, of course, excludes prescribing upon any one symptom, as has become somewhat a fashion. These single symptoms are very useful in indicating the drug or drugs one should study. After this brief review of Hahnemann's teaching and of his practice, we believe it is correct to state, as we have already done, that *the characteristics of any drug are the particular effects of that drug which no other remedy produces in a precisely similar manner.* E. J. L.

